# SARS-CoV-2-specific humoral immunity in a Norwegian cohort between 2020 and 2023

**DOI:** 10.1186/s12916-025-04171-2

**Published:** 2025-06-03

**Authors:** Marjut Sarjomaa, Kristine Karlsrud Berg, Keson Jaioun, Yngvar Tveten, Hege Kersten, Harald Reiso, Randi Eikeland, Carina Thilesen, Svein Arne Nordbø, Ingeborg S. Aaberge, Neil Pearce, Anne Kristin Moeller Fell

**Affiliations:** 1 Department of Infection Control, Telemark Hospital Trust, Ulefossvegen 55, 3710 Skien, Norway; 2 Department of Community Medicine and Global Health, University of Oslo, Oslo, Norway; 3 Department of Medical Microbiology, Sørlandet Hospital Trust, Kristiansand, Norway; 4 Department of Research, Telemark Hospital Trust, Skien, Norway; 5 Department of Occupational and Environmental Medicine, Telemark Hospital Trust, Skien, Norway; 6 Department of Clinical Microbiology, Telemark Hospital Trust, Skien, Norway; 7 Department of Geriatric Medicine, Institute of Clinical Medicine, Faculty of Medicine, University of Oslo, Oslo, Norway; 8 The Norwegian Advisory Unit On Tick-Borne Diseases, Sørlandet Hospital Trust, Arendal, Norway; 9 Department of Health and Sport Science, Institute of Health and Nursing Science, University of Agder, Grimstad, Norway; 10 Unilabs Laboratory Medicine, Skien, Norway; 11 Department of Medical Microbiology, St. Olavs Hospital, Trondheim University Hospital, Trondheim, Norway; 12 Norwegian University of Science and Technology, Trondheim, Norway; 13 Norwegian Institute of Public Health, Oslo, Norway; 14 London School of Hygiene and Tropical Medicine, London, UK

**Keywords:** COVID-19, SARS-CoV-2, Cohort studies, Vaccines, Antibodies, Immunity

## Abstract

**Background:**

We have previously reported on natural humoral immunity against severe acute respiratory syndrome coronavirus 2 (SARS-CoV-2) in a Norwegian cohort between 2020 and 2021. In this study, we evaluated long-term humoral (including vaccination-induced) immunity in the same cohort and assessed predictors of high antibody levels against spike protein, as well as the persistence of antibodies against the virus spike and nucleocapsid proteins.

**Methods:**

Vaccination data and antibody levels against the spike and nucleocapsid proteins were collected at 12 (only in infected participants) and 24 months (in both infected and uninfected participants) after the participants’ first polymerase chain reaction (PCR) tests for the virus. Antibody levels against spike protein at 24 months were categorized as high or low based on the 50th percentile. Possible predictors of high antibody levels against spike protein were examined using univariate and multivariate logistic regression models.

**Results:**

Of 1119 original participants (400 PCR + and 719 PCR −), 574 responded to our questionnaires and were invited to antibody measurements (median age: 51 years; women: 59%). Vaccination data showed that 11% were fully immunized, and 85% were booster-immunized at 24 months. Antibody levels were evaluated in 72% (287/400) of the PCR + participants at 12 months and 58% (233/400) at 24 months. At 12 and 24 months, we observed that 97% (278/287) and 100% (233/233), respectively, still had antibodies against the spike protein, and 86% (248/287) and 95% (221/233), respectively, against the nucleocapsid protein. Antibody levels were also evaluated in 34% (247/719) of those in the PCR − group, which revealed that 99.5% and 69% had detectable antibodies against spike and nucleocapsid proteins, respectively, at 24 months. Irrespective of pre-vaccination SARS-CoV-2 infection status, the booster-immunized participants were 3.7 × more likely to have high antibody levels against spike protein vs the non-booster-immunized ones. Those aged > 60 years had the highest median antibody levels against the spike protein and were more likely to be booster-immunized.

**Conclusions:**

Our findings highlight the benefits of booster vaccinations for humoral immune responses. Long-term antibody levels against the SARS-CoV-2 spike protein were higher in booster-immunized participants vs the non-booster-immunized, irrespective of pre-vaccination infection status.

Trial registration.

146,469: The COVID-19 study in Telemark and Agder—COVITA. ClinicalTrials.gov ID: NCT04514003.

**Supplementary Information:**

The online version contains supplementary material available at 10.1186/s12916-025-04171-2.

## Background

In February 2025, the World Health Organization reported 777,519,152 cumulative coronavirus disease (COVID-19) cases, 7,090,776 deaths related to the disease, and 13.64 billion COVID-19 vaccine doses administered worldwide [1]. The first COVID-19 messenger ribonucleic acid (mRNA) and vector-based vaccines were made available in late December 2020 [2]. Immunization with these vaccines induced high levels of neutralizing antibodies against the spike (S) protein of the severe acute respiratory syndrome coronavirus 2 (SARS-CoV-2) virus, while infection with the virus-induced antibodies against both S and nucleocapsid (N) proteins [3, 4]. During the early phases of the COVID-19 pandemic, some studies reported that SARS-CoV-2 infection induced humoral immunity that persisted for months [5] before gradually declining [6, 7]. Primary and booster vaccination protected primarily against severe illness and death—as well as (to a much lower degree) against mild infection and viral transmissibility [8–10]. Furthermore, new variants of concern (VOCs), particularly the omicron variant, evaded the protective humoral immunity conferred by prior infection or vaccination, resulting in lower vaccine effectiveness [10–12]. Antibody levels also waned over time following vaccination [8, 9]. In the post-pandemic era, a knowledge gap persists regarding long-term immunity to SARS-CoV-2 and VOCs following infection (i.e., natural immunity), vaccination (i.e., vaccine-induced humoral immunity), and both challenges (i.e., hybrid immunity). A meta-analysis from 2024 showed that individuals with hybrid immunity achieved by infection and booster vaccination had higher level of protection against reinfection compared to individuals with complete or incomplete vaccination [13].

Hybrid immunity has also shown higher and broader humoral immunity compared to infection or vaccination alone [8, 11, 14]. Recommendations concerning vaccination following SARS-CoV-2 infection and COVID-19 have therefore been modified both during and after the pandemic, owing to an evolving understanding of the duration of antibody persistence following initial infection, viral escape from VOCs, and individuals at higher risk of infection [2].

Several studies have assessed the persistence of antibodies following SARS-CoV-2 infection and COVID-19 in both the pre-vaccine and pre-omicron periods [5, 7, 15, 16]. Several studies on hybrid immunity have included specific populations, such as seriously ill patients or healthcare workers [17, 18]. In contrast, few studies with long-term follow-up periods have included individuals with either SARS-CoV-2 infection or COVID-19, reflecting the whole illness spectrum from asymptomatic to seriously ill patients [17, 18].

We conducted a 2-year follow-up study consisting of participants with SARS-CoV-2 infection or COVID-19. We assessed long-term natural, hybrid, and vaccine-induced humoral immunity in two cohorts of adult participants who tested positive for SARS-CoV-2 infection via polymerase chain reaction (PCR)—constituting the “PCR + ” group—as well as those who tested negative, constituting the “PCR − ” group. We also assessed possible predictors of high antibody levels against S protein at 24 months and characterized antibody persistence against S and N proteins at 12 and 24 months following the initial PCR tests performed in 2020.

### Definitions used in the study

The PCR + participants had positive SARS-CoV-2 real-time PCR results at a COVID-19 testing center or hospital upon their inclusion in 2020, while the PCR − ones returned negative results for the same test. The initial PCR test in 2020 established the criteria for being in the PCR positive or negative group; subsequent testing had no impact on the participants’ initial group assignment. SARS-CoV-2 infections at designated “T1” (12 months) and “T2” (24 months) time points were defined as self-reported positive PCR or rapid antigen test results at the respective time points. The humoral immune response following primary SARS-CoV-2 infection and one vaccine dose has previously been shown to be equivalent to or higher than that conferred by two doses in infection-naive adults [14, 19, 20]. Therefore, we defined fully immunized as PCR + participants who had been vaccinated with one primary vaccine dose, PCR − participants who had been vaccinated with two vaccine doses, and PCR − participants who reported SARS-CoV-2 infection at T2 and were immunized with one vaccine dose:

- Comirnaty (Pfizer/BioNTech; BNT162b2, USA/Germany) alone or combined with Spikevax (Moderna; mRNA-1273, USA/Switzerland)
- Two heterologous vaccines with the adenovector-based vaccine Vaxzevria (AstraZeneca; ChAdOx nCoV-19; AZD1222, USA/UK) combined with Comirnaty or Spikevax

No significant differences were detected in terms of the antibody levels between the homologous and heterologous vaccinated groups at T2; therefore, these groups were combined for the rest of the study. The booster-immunized group was defined as the participants who were fully immunized and had also received one or more booster vaccine doses.

## Methods

Our aim was to follow-up initially SARS-CoV-2-infected (PCR +) and SARS-CoV-2-naïve (PCR −) adults residing in South-Eastern Norway with questionnaires and evaluate levels of antibodies against the SARS-CoV-2 S and N proteins at 12 and 24 months after their initial PCR tests in 2020. We then assessed the persistence and possible predictors of high antibody levels against S protein.

### Study design and data sources

This multicenter cohort study included individuals from all hospitals, municipal laboratories, and test centers in the region. The cohort was chosen from PCR + and PCR − adults (≥ 18 years of age) living in South-Eastern Norway (Agder and Telemark counties) who were reachable by phone during the inclusion period (between February 28 and December 17, 2020—between the first and second waves of the pandemic). Participants who could not answer the questionnaire in Norwegian were excluded.

We included baseline data (defined as the time of the first questionnaire) for the PCR + participants, 3–5 months after the first PCR test, and from a shorter follow-up questionnaire at 12 months (interquartile range [IQR]: 9–12 months), defined as T1, as well as at 24 months (IQR: 23–26 months), defined as T2. T1 ranged between January 21, 2021 and February 9, 2022, and T2 between March 23, 2022 and January 10, 2023. Antibody and questionnaire follow-up data for the PCR − participants was available at T2. The median antibody levels against S protein at T1 and T2 were also stratified by age groups for the PCR + participants with paired measurements (*n* = 193/400). We have previously reported on natural immunity in this cohort between 2020 and 2021, as well as risk factors for SARS-CoV-2 infection [5, 21].

### Study population

The criteria for SARS-CoV-2 testing changed in Norway over the study period but there were no differences between the PCR + and PCR − participants. In the first wave of the pandemic in Norway, the symptomatic patients were tested using PCR. In the second wave, also close contacts and asymptomatic individuals were tested during the outbreaks [22]. We used the results of the first PCR test for each participant. We aimed to include all of the eligible PCR + and PCR − participants in a 1:2 ratio, matched according to the time of PCR testing and geographical location during the study period.

### Laboratory methods

Antibodies against the SARS-CoV-2 S and N proteins were tested in serum samples collected from the PCR + participants at 12 and 24 months following their initial PCR tests and at 24 months following their initial PCR tests for the PCR − ones.

The serum samples were prepared from whole blood centrifuged for 10 min at 3000 rpm and stored at − 80 °C for further analysis. All antibody analyses were performed using a Cobas 801 fully automated system (Roche Diagnostics, Mannheim, Germany). An Elecsys Anti-SARS-CoV-2 S electrochemiluminescence immunoassay (Roche) was used to quantitatively measure antibody levels against the S protein. This immunoassay quantifies total antibodies using a recombinant protein that represents the receptor-binding domain of the S protein in a double-antigen sandwich assay format. All tests and analyses were performed according to the manufacturers’ instructions. The cutoff value for units per milliliter (U/mL) was > 0.8 U/mL. Owing to the exceptionally high concentrations of antibodies against the S protein at 24 months, samples of > 250 U/mL were diluted several times and re-measured. Dilutions of 1:10, 1:50, and 1:400 yielded a measuring range of 0.4–100,000 U/mL. The Elecsys Anti-SARS-CoV-2 electrochemiluminescence immunoassay was used to detect antibodies against the N protein. A double-antigen sandwich assay was used to assess antibodies against the S protein, with the recombinant protein being represented by the N protein. Antibodies against the N protein were analyzed and interpreted according to the manufacturer’s instructions, with a cutoff index of > 1 being considered positive.

### Vaccination status

A unique personal identification number linked each participant to the Norwegian Immunization Registry (SYSVAK), providing information regarding the type and date of COVID-19 vaccine doses received between January 2021 and January 2023. The SYSVAK represents a national electronic registry that records every vaccine received by individuals in Norway [23].

### Data collection

#### Questionnaires

We used questions from the Norwegian Institute of Public Health’s COVID-19 questionnaire, the Telemark study’s questionnaire [24, 25], as well as several questions that were unique to this study. Sociodemographic data, such as age, sex, education, and income, and lifestyle factors, such as body mass index (BMI), smoking status, symptoms at baseline, and pre-existing comorbidities, were obtained from a self-reported questionnaire. Symptom and comorbidity scores were calculated by adding the number of symptoms from 0 to 13 (coughing, running or stuffy nose, sore throat, pain upon swallowing, dyspnea, headache, fever, fever with chills or sweating, pain in the stomach, nausea or diarrhea, impaired sense of smell or taste, myalgia, and dizziness) or comorbidities from 0 to 9 (asthma, chronic obstructive pulmonary disease, other lung diseases, cancer, heart disease, hypertension, diabetes, musculoskeletal disease, or any other disease) for each participant. Each symptom and comorbidity was considered equally. All positive PCR tests and commercially available SARS-CoV-2 antigen tests reported by the participants during the follow-up period were recorded. COVID-19 vaccination status was reported based on the date and type of vaccine received.

### Statistical analysis

We did not conduct statistical power calculations because it was unclear in advance how many participants would be eligible for inclusion over the study period. The normality of the distribution of all continuous variables was assessed using the Shapiro–Wilk test. Medians and IQRs were used to express non-normally distributed variables. Wilcoxon rank-sum and Kruskal–Wallis tests were used to compare differences in median dispersions between groups, as appropriate. Dunn’s test was applied with Bonferroni correction for multiple comparisons. Categorical variables were expressed as frequencies and percentages and compared using Pearson’s chi-squared or Fisher’s exact tests, as appropriate.

Antibody levels against the S protein at 24 months were categorized as high or low, based on the 50th percentile. The cutoff value for antibodies against the S protein was 9890 U/mL. Possible predictors of high antibody levels against the S protein at 24 months were studied using both univariate and multivariate logistic regression models. Regression analysis results were calculated and presented as odds ratios (ORs) with associated 95% confidence intervals (CIs). All statistical analyses were performed using STATA version 18.0 (StataCorp, College Station, TX, USA). Statistical significance was set at *p* < 0.05.

Our returned questionnaires had missing data, ranging between 0.4 and 7.3% for certain questions. All variables with missing data were assessed and found to be random, so imputation was not performed for the missing data.

### Ethics

Participation in the study was voluntary. After verbally agreeing to participate at the time of the baseline survey, all of the participants also provided written informed consent prior to being included. The participants then completed the first questionnaire on paper. Shorter online follow-up questionnaires were then completed at T1 and T2 for the PCR + participants and at T2 for PCR − ones. The PCR − participants initially only consented to participate at the time of the baseline survey; however, following a revised written agreement, they were later recruited for the 24-month follow-up survey as well. The study was approved by the Regional Committee for Medical and Health Research Ethics of South-East Norway (ID: 146,469).

### Patient and public involvement

Two user representatives were involved, according to the Norwegian National Guidelines for User Involvement in Health Research (May 2018). They played an important role in all phases of the project—particularly regarding the development and testing of the questionnaires. They helped us to better understand the patients’ points of view and provided valuable feedback concerning our study protocol, methods, information, consent forms, questionnaires, and dissemination of the results.

## Results

### Characterization of the study cohort

**Fig. 1:**
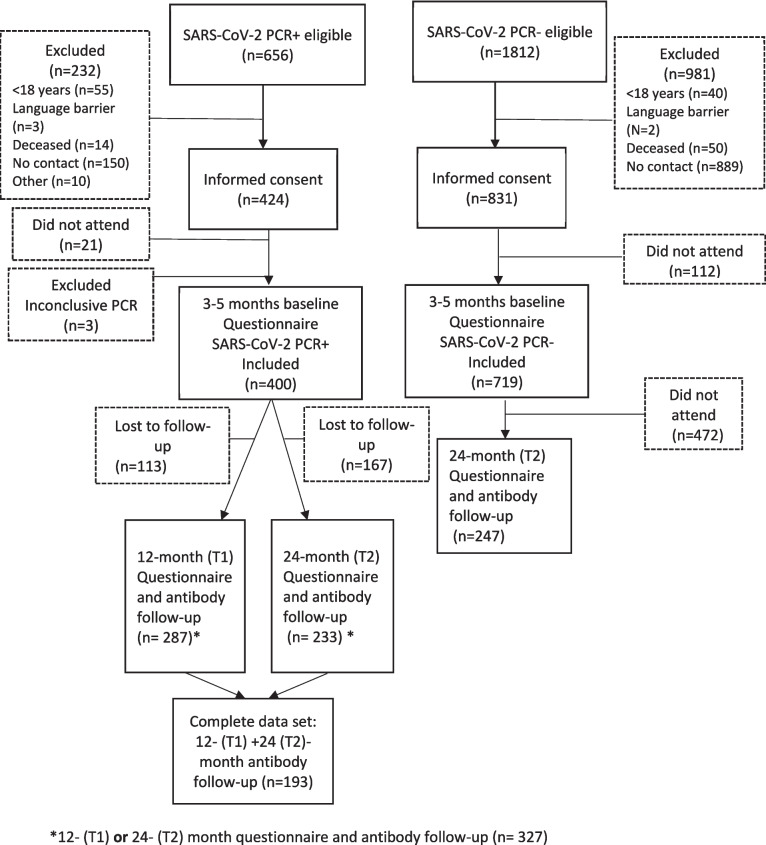
Flow chart that shows inclusion and exclusion of the participants and follow-ups

**Fig. 2:**
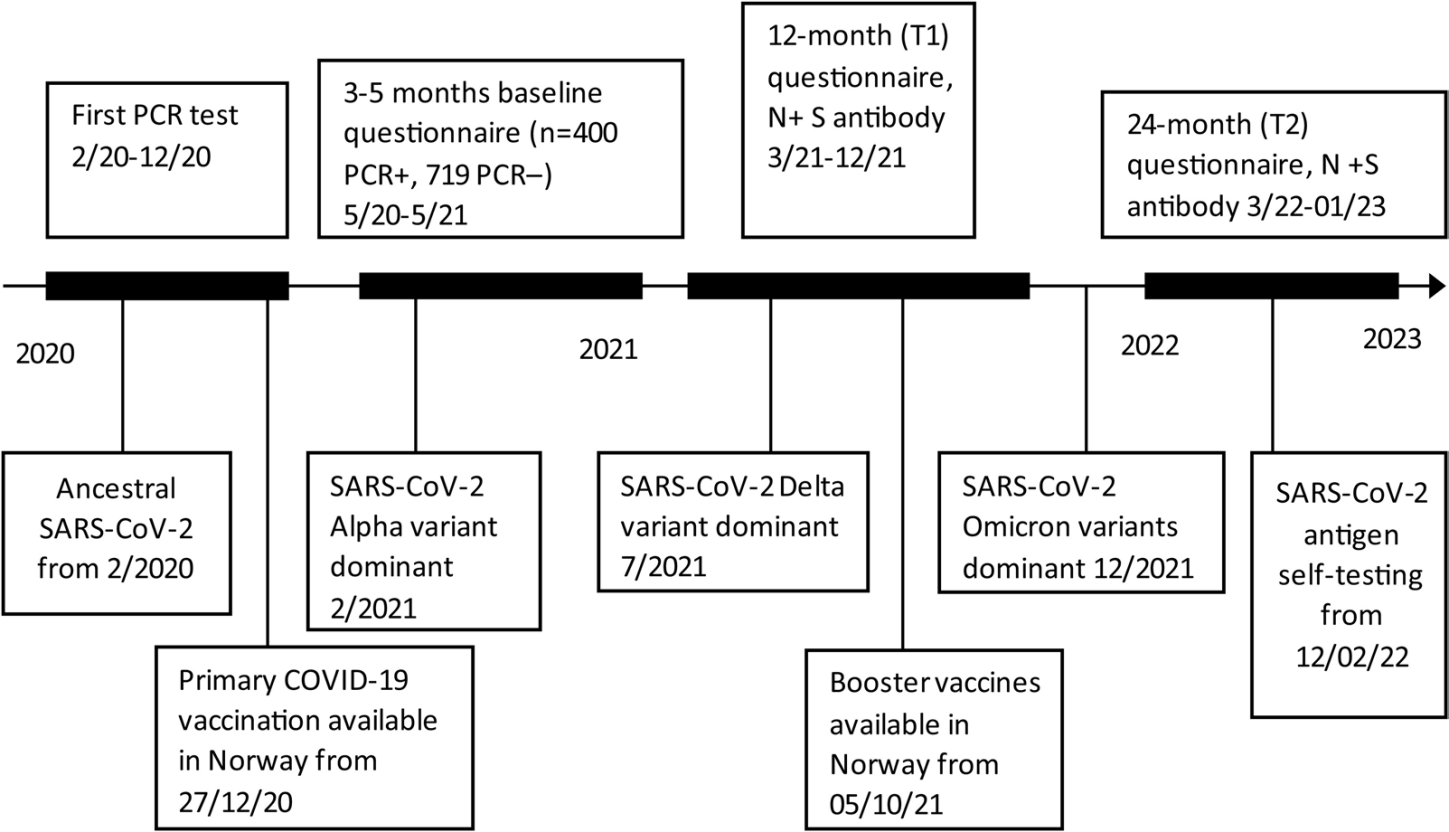
Timeline with different virus variants, vaccination, questionnaires, and antibody measurements for the study

Of 2468 total eligible participants, 1119 were included. Among these, 400 were PCR + (400/656) while 719 were PCR − (719/1812). The participants were recruited between February 28 and December 17, 2020 (Fig. 1). Both symptomatic and asymptomatic participants were included reflecting the pandemic situation in the general population in Norway. Only 5% (58/1119) were asymptomatic when the PCR test was taken and 6% (22/400) of the PCR + participants were hospitalized [5]. The symptomatic PCR − participants likely had symptoms related to other infections. The median age of this cohort was 51 years (IQR, 39–61) at baseline (Table 1). Females made up 50% (163/327) of the PCR + participants and 71% (175/247) of the PCR − . Of the initially PCR + participants, 72% (287/400) had measured antibodies against the N and S proteins at T1. At T2, 58% (233/400) of the PCR + participants and 34% (247/719) of the PCR − ones had measured antibodies against the S and N proteins. Of the PCR + participants, 48% (193/400) had their antibodies measured at both the T1 and T2 time points. The ancestral, alpha, delta, and omicron (beginning in December 2021) SARS-CoV-2 variants dominated in Norway at T1, while only omicron variants were present at T2 [22]. Figure 2 shows the emergence of SARS-CoV-2 variants in Norway, the Norwegian SARS-CoV-2 vaccination timeline, and the serum samples and questionnaires collected over the study period.

**Table 1:** Characteristics of the study population at the baseline survey (3–5 months following the first PCR test) and the follow-ups

Characteristic	Total (*n* = 574)^*^	PCR + (*n* = 327)	PCR − (*n* = 247)
Age in years, median (IQR)	50.5 (39–61)	50 (38–59)	51 (40–62)
Age (year) in categories, *n* (%)			
18–30	53 (9.2)	39 (11.9)	14 (5.7)
31–40	105 (18.3)	56 (17.1)	49 (19.8)
41–50	129 (22.5)	77 (23.6)	52 (21.0)
51–60	138 (24.0)	76 (23.2)	62 (25.1)
> 60	149 (26.0)	79 (24.1)	70 (28.3)
Sex, female (%)	338 (58.9)	163 (49.8)	175 (70.8)
BMI in kg/m^2^, median (IQR), (*n* = 556)	25.7 (23.3–29.0)	25.7 (23.3–28.7)	25.7 (23.3–29.3)
BMI in category, kg/m^2^, *n* (%)			
Underweight	7 (1.2)	4 (1.2)	3 (1.21)
Normal weight	232 (40.4)	133 (40.7)	99 (40.1)
Overweight	215 (37.5)	119 (36.4)	96 (38.9)
Obese	102 (11.8)	57 (17.4)	45 (18.2)
Missing	18 (3.1)	14 (4.3)	4 (1.6)
Education			
Primary and secondary school	54 (9.4)	33 (10.9)	21 (8.5)
High school and certificate	179 (31.2)	116 (35.5)	63 (25.5)
University	332 (57.8)	170 (52.0)	162 (65.6)
Missing	9 (1.6)	8 (2.4)	1 (0.4)
Income			
< 500,000 NOK	93 (16.2)	50 (15.3)	43 (17.4)
500,000–1,000,000 NOK	240 (41.8)	129 (39.5)	111 (44.9)
≥ 1,000,000 NOK	215 (37.5)	124 (37.9)	91 (36.8)
Missing	26 (4.5)	24 (7.3)	2 (0.8)
Smoking status, *n* (%)			
Never smoker	294 (51.2)	177 (54.1)	117 (47.4)
Past smoker	179 (31.2)	101 (30.9)	78 (31.6)
Occasional and daily smoker	72 (12.5)	31 (9.5)	41 (16.6)
Missing	29 (5.1)	18 (5.5)	11 (4.4)
Comorbidities, *n* (%)			
Asthma	90 (15.7)	51 (15.6)	39 (15.8)
COPD	13 (2.3)	6 (1.8)	7 (2.8)
Other chronic lung disease	22 (3.8)	13 (4.0)	9 (3.6)
Cancer	14 (2.4)	6 (1.8)	8 (3.2)
Heart disease	37 (6.5)	16 (4.9)	21 (8.5)
Hypertension	67 (11.7)	33 (10.1)	34 (13.8)
Diabetes	31 (5.4)	18 (5.5)	13 (5.3)
Musculoskeletal disease	31 (5.4)	13 (4.0)	18 (7.3)
Any other disease	93 (16.2)	44 (13.5)	49 (19.8)
Comorbidity score^**^, median (IQR)	0 (0–1)	0 (0–1)	0 (0–1)
Symptoms at first PCR test			
Cough	258 (44.9)	149 (45.6)	109 (44.1)
Running nose	225 (39.2)	97 (29.7)	128 (51.8)
Stuffy nose	205 (35.7)	97 (29.7)	108 (43.7)
Sore throat	256 (44.6)	126 (38.5)	130 (52.6)
Pain upon swallowing	108 (18.8)	37 (11.3)	71 (28.7)
Dyspnea	229 (39.9)	158 (48.3)	71 (28.7)
Headache	307 (53.5)	201 (61.5)	106 (42.9)
Fever	295 (51.4)	210 (64.2)	85 (34.4)
Fever with chills or sweating	180 (31.4)	130 (39.8)	50 (20.2)
Abdominal pain, nausea, or diarrhea	132 (23.0)	90 (27.5)	42 (17.0)
Impaired sense of smell or taste	244 (42.5)	207 (63.3)	37 (15.0)
Myalgia	252 (43.9)	179 (54.7)	73 (29.6)
Dizziness	183 (31.9)	132 (40.4)	51 (20.6)
Symptom score^**^, median (IQR)	5 (3–7)	6 (4–8)	4 (2–6)
Follow-up visit, months^†^, median (IQR) (min–max)			
Month 12 (T1)	10 (9–12) (7–16)		
Month 24 (T2)	24 (23–26) (17–33)		

*PCR* polymerase chain reaction, *IQR* interquartile range, *BMI* body mass index, *NOK* Norwegian kroner, *One NOK* 0.087 Euro, *COPD* chronic obstructive pulmonary disease

^*^Includes the initially SARS-CoV-2 PCR+ participants who had measured antibodies at 12 (T1) or 24 months (T2) following their first PCR tests, and the initially SARS-CoV-2 PCR– participants who measured the antibodies at 24 months

^**^Total symptom and comorbidity scores were calculated by adding the number of symptoms or comorbidities for each participant

^†^Time after first PCR test

### Duration and level of antibodies against spike and nucleocapsid protein

Additional file 1: Table 1 shows the homologous and heterologous vaccination groups at T2, which were combined into a single vaccination group. Of the PCR + participants, 97% (278/287) had detectable antibodies against the S protein at T1 and 100% at T2 (233/233) (Table 2). Of the PCR + participants, 86% (248/287) had antibodies against the N protein at T1 and 95% (221/233) at T2. Antibodies against the S protein were detectable for almost all (246/247) of the PCR − participants at T2. Antibodies against the N protein were detectable for 69% (171/247) of the PCR − participants at T2. Among the PCR + and PCR − participants, 83% (272/327) and 88% (217/247), respectively, were booster-immunized at T2.

**Table 2:** Vaccination status and antibody levels against the SARS-CoV-2 spike (S) and nucleocapsid (N) proteins measured at 12 (T1) and 24 months (T2) among the initially SARS-CoV-2-infected (PCR +) and SARS-CoV-2-naïve (PCR −) participants

Characteristics	Total (*n* = 574)^*^	PCR + (*n* = 327)	PCR − (*n* = 247)	*p* value^**^
Vaccination groups^†^				
Booster-immunized	489 (85.2)	272 (83.2)	217 (87.9)	0.248
Fully immunized	65 (11.3)	43 (13.1)	22 (8.9)	
Unvaccinated	18	10 (3.1)	8 (3.2)	
Missing data	2 (0.4)	2 (0.6)	0	
Participants who measured antibodies, *n*				
T1, *n*	287	287	na	
T2, *n*	480	233	247	
S antibodies (U/mL)				
T1, positive (> 0.8), *n* (%)^‡^	278 (96.9)	278 (96.9)	na	
T2, positive (> 0.8), *n* (%)^¶^	479 (99.8)	233 (100)	246 (99.6)	
T1, median (IQR)^‡^	208 (57–1586)	208 (57–1586)	na	
T2, median (IQR)^¶^	9890 (4512–27,910)	9011 (5237–21,362)	11,721 (3892–32,940)	0.228
N antibodies (COI)				
T1, positive (> 1.0), *n* (%)^‡^	248 (86.4)	248 (86.4)	na	
T2, positive (> 1.0), *n* (%)^¶^	392 (81.7)	221 (94.9)	171 (69.2)	
T1, median (IQR)^‡^	12.3 (3.1–41.6)	12.3 (3.1–41.6)	na	
T2, median (IQR)^¶^	12.8 (2.3–53.8)	33.3 (5.5–92.8)	6.2 (0.1–22.6)	** < 0.001**

*COI*, cutoff index; *na*, not available.

^*^Includes the initially SARS-CoV-2 PCR + participants who measured antibodies at 12 (T1) or 24 months (T2) following their first PCR test and the initially SARS-CoV-2 PCR − participants who measured antibodies at 24 months.

^**^Statistical significance is shown in bold.

^†^Fully immunized: PCR + and 1 vaccine dose, PCR − and two vaccine doses, or PCR − and infection reported at 24 months and one vaccine dose; booster-immunized: PCR + and ≥ 2 vaccine doses, PCR − with ≥ 3 vaccine doses, or PCR − and infection reported at 24 months and two vaccine doses.

^‡^Two hundred eighty-seven PCR + participants measured the antibodies at 12 months.

^¶^Four hundred eighty PCR + and PCR − participants measured the antibodies at 24 months (PCR + *n* = 233; PCR − *n* = 247).

For PCR + participants, no association between symptom score and antibody levels against S or N protein were detected. The median antibody levels at 24 months were stratified by booster-immunization status (Table 3)*.* Median antibody levels for S antibodies were higher for booster-immunized than non-booster-immunized PCR + and PCR − participants at T2. Median N antibody levels for non-booster-immunized PCR − participants were higher than for booster-immunized PCR − participants at T2.

**Table 3:** Median antibody levels against spike (S) and nucleocapsid (N) protein at 24 months (T2) among the initially SARS-CoV-2-infected (PCR +) and SARS-CoV-2-naïve (PCR −) participants stratified by booster-immunization status

	Booster-immunized	Non-booster-immunized	*p* value^*^
PCR +			
*n*	199	33	
S antibody (U/mL) at T2, median (IQR)	9665.0 (5979.0–23,747.0)	4084.0 (3037.0–7636.0)	** < 0.001**
N antibody (COI) at T2, median (IQR)	33.2 (5.0–88.4)	43.1 (14.6–118.0)	0.232
PCR −			
*n*	217	30	
S antibody (U/mL) at T2, median (IQR)	15,809.0 (4480–33,796.0)	5283.5 (124.0–16,690.0)	**0.001**
N antibody (COI) at T2, median (IQR)	5.0 (0.1–20.9)	17.6 (2.8–93.9)	**0.009**

*PCR*, polymerase chain reaction; *IQR*, interquartile range; *COI*, cutoff index.

^*^Statistical significance is shown in bold.

### Possible predictors for high antibody levels against spike and nucleocapsid protein

The participants who had their antibodies measured and answered the questionnaire at T2 (*n* = 440) were categorized into two groups based on low (< 50th percentile) and high (> 50th percentile) antibody levels against S protein (ST2High). The S protein antibody level was 9890 U/mL at the 50th percentile. The booster-immunized participants were 3.7 × more likely to be in the ST2High group than the fully immunized and unvaccinated ones. Participants with university degrees were 2.2 × more likely to be in the ST2High group vs those with primary or secondary educations (Table 4). Those with a university degree were more likely booster-immunized (87%) than those with only primary or secondary school education (83%).

**Table 4:** Possible predictors of high antibody levels against the SARS-CoV-2 S protein at T2 (ST2High) among the initially SARS-CoV-2 PCR + and PCR − participants, compared to low levels (*n* = 440), assessed using univariate and multivariate analyses adjusted for all variables

ST2High^*^	Univariate analysis		Multivariate analysis	
	OR (95% CI)	*p* value^**^	OR (95% CI)	*p* value^**^
Age group (18– > 60 years)				
18–30	ref		ref	
31–40	1. 218 (0.506–2.932)	0.660	0.996 (0.389–2.552)	0.994
41–50	1.328 (0.567–3.110)	0.514	1.034 (0.419–2.554)	0.942
51–60	1.694 (0.736–3.899)	0.215	1.343 (0.548–3.290)	0.548
> 60	2.121 (0.925–4.864)	0.076	1.430 (0.577–3.541)	0.440
Sex				
Male	ref		ref	
Female	0.611 (0.416–0.896)	**0.012**	0.678 (0.448–1.026)	0.066
BMI (kg/m^2^)				
18.5–24.999	ref		ref	
< 18.5	0.617 (0.550–6.930)	0.696	0.891 (0.685–11.593)	0.930
25–29.999	1.475 (0.968–2.248)	0.071	1.402 (0.891–2.205)	0.144
> 30	1.100 (0.659–1.838)	0.715	1.087 (0.625–1.892)	0.767
Education				
Primary and secondary school	ref		ref	
High school and certificate	1.857 (0.880–3.918)	0.104	2.577 (0.809–8.215)	0.109
University	2.124 (1.047–4.311)	**0.037**	2.539 (1.165–10.756)	**0.026**
Income				
< 500,000 NOK	ref		ref	
500,000–1,000,000 NOK	1.351 (0.777–2.352)	0.287	0.806 (0.341–1.908)	0.624
> 1,000,000 NOK	1.427 (0.813–2.505)	0.215	0.908 (0.364–2.264)	0.837
Smoking status				
Never smoker	ref		ref	
Past smoker	1.189 (0.791–1.789)	0.405	0.748 (0.399–1.399)	0.363
Occasional + daily smoker	0.917 (0.508–1.654)	0.773	1.055 (0.434–2.567)	0.906
Symptom score	0.976 (0.919–1.036)	0.418	1.010 (0.922–1.106)	0834
Comorbidity score	1.099 (0.910–1.326)	0.327	1.124 (0.826–1.529)	0.457
Immunization status				
Unvaccinated	ref		ref	
Fully immunized				
Booster-immunized	4.347 (2.233–8.465)	** < 0.001**	3.672 (1.855–7.271)	** < 0.001**

*OR*, odds ratio; *CI*, confidence interval; *BMI*, body mass index; *NOK*, Norwegian kronor. One NOK = 0.087 Euro.

^*^At 24 months (T2), the participants were categorized into two groups based on antibody levels against the SARS-CoV-2 spike protein (S), based on whether their levels were above (high) or below (low) the 50th percentile value.

^**^Statistical significance was defined as *p* < 0.05.

**Fig. 3:**
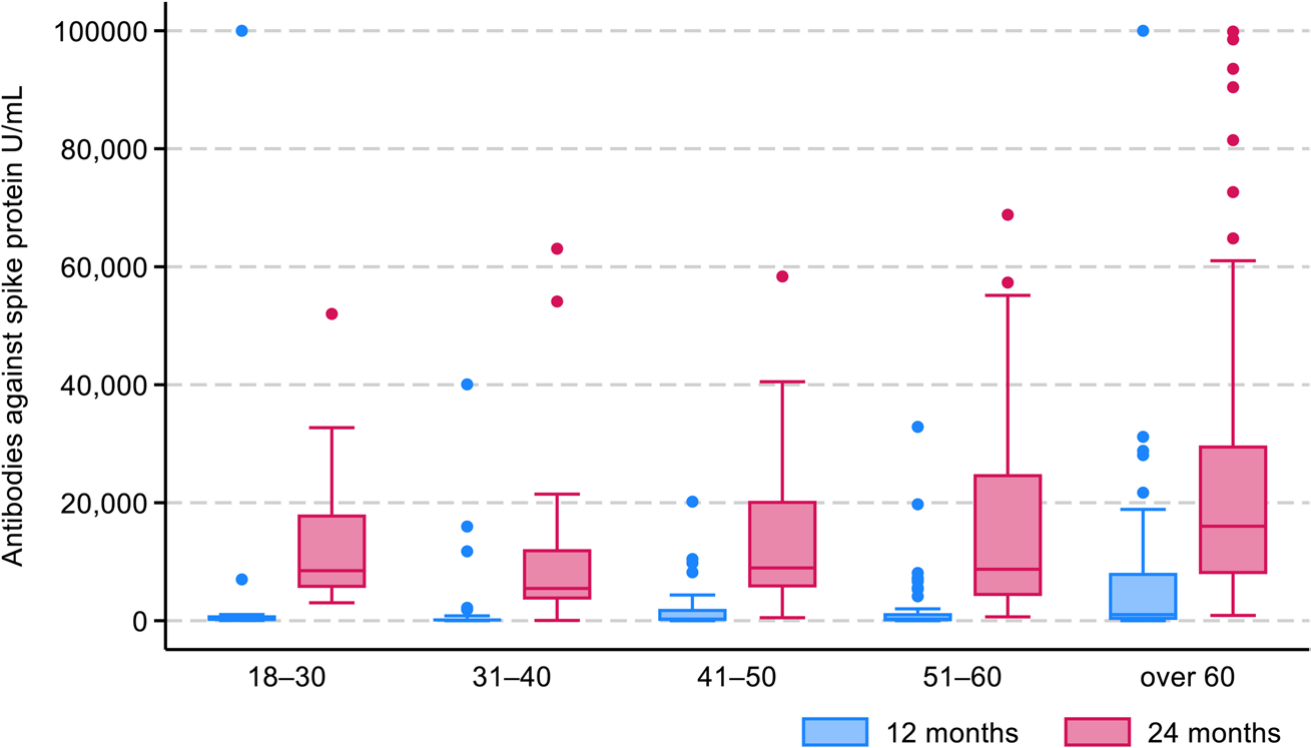
Box plot of median antibody values against the SARS-CoV-2 spike protein at 12 (T1) and 24 (T2) months following the first PCR test among PCR + participants with paired measurements (193/400), stratified by age group

In comparison to PCR + females, PCR + males had significant higher antibody levels against S protein at T2 and N protein at T1 (Additional file 2: Table 2). PCR + males were older (median age: 51.5 years vs 47.0 years, *p* < 0.001) and had higher BMI (median: 26.6 kg/m^2^ vs 24.8 kg/m^2^, *p* = 0.002) than PCR + females. PCR + participants aged > 60 years had the highest median antibody levels against S protein at T2 (Fig. 3).

### Antibody levels against S protein and immunization

**Fig. 4:**
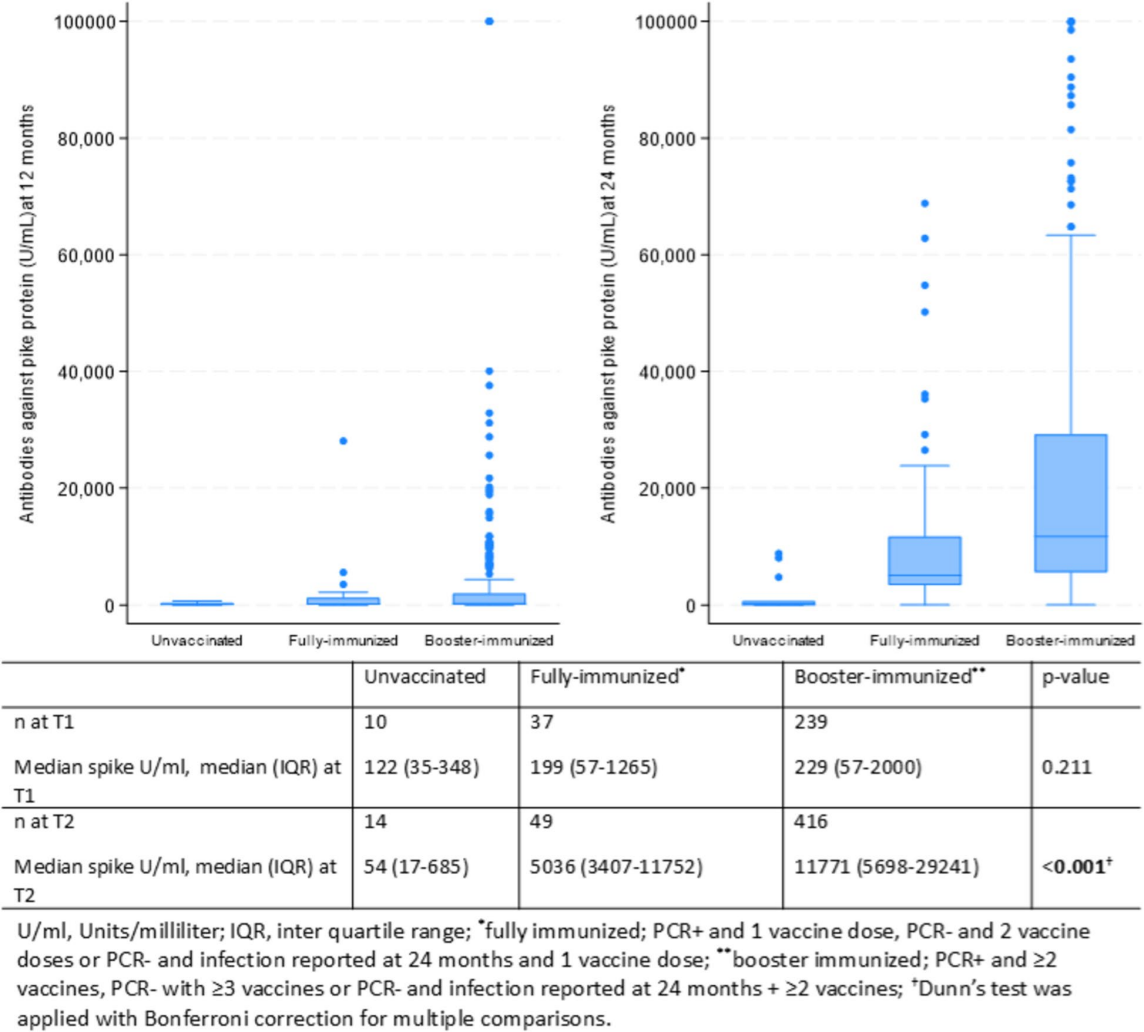
Box plot and table showing antibody levels against the SARS-CoV-2 spike protein at 12 (left) and 24 months (right) following initial PCR testing among initially SARS-CoV-2-infected and SARS-CoV-2-naïve participants, stratified by immunization status

Comparison of median antibody levels against S protein among the unvaccinated, fully immunized, and booster-immunized participants at T1 and T2 showed that the booster-immunized participants had the highest antibody levels at T2 (Fig. 4). The antibody levels against S protein at T2 for PCR + (*n* = 232) and PCR − (*n* = 274) participants were stratified by vaccination coverage (Additional file 3: Fig. S1). The highest levels for antibodies against S protein were detected for booster-immunized participants irrespective of the pre-vaccination SARS-CoV-infection status. The age group distribution and the immunization status in percentage showed that the booster-immunization increased with older age and was highest for those > 60 years (Additional file 4: Fig. S2).

## Discussion

### Duration and level of antibodies against spike and nucleocapsid protein

In the present study, the persistence and possible predictors for long-term natural, hybrid, and vaccine-induced humoral immunity were assessed using a cohort of mainly non-hospitalized participants from the first two pandemic waves in Norway in 2020. At T2, 83% and 88% of the PCR + and PCR − individuals, respectively, were booster-immunized. Furthermore, antibodies against S protein persisted up to 24 months among fully and booster-immunized participants irrespective of the pre-vaccination SARS-CoV-2 infection status. These findings are in line with other studies, which showed circulating antibodies against S protein up to 15–27 months after vaccination for those with and without previous infection [17, 18]. Of the PCR + participants, 86% had antibodies against N protein at T1 and 95% at T2; for PCR − participants they were detectable among 69%. As reported in a study assessing HCW in USA, immunoassays targeting the N protein can detect previous infection in populations vaccinated with spike protein vaccines, but the study showed seroreversion of N total immunoglobulin by 18 months postinfection [26]. In our study, the high percentage of antibodies against N protein at 24 months might reflect a high number of reinfections during follow-up.

### Possible predictors for high antibody levels against spike and nucleocapsid protein

The median antibody levels for S antibodies were higher for booster-immunized than non-booster-immunized PCR + and PCR − participants at T2. Furthermore, when we categorized the participants into low and high antibody levels against S protein, booster-immunized were 3.7 × more likely to have high antibody levels than non-booster-immunized at T2. This finding was not dependent on the prior SARS-CoV-2 infection status. This demonstrates that in this cohort the humoral immunity was enhanced with sustained immunization for both SARS-CoV-2-infected and SARS-CoV-2-naïve individuals at T2. There are few epidemiological cohort studies assessing longtime follow-up of antibody levels by different vaccination coverages for previously SARS-CoV-2-infected patients and SARS-CoV-2-naïve participants. In agreement with our study, a 10-month follow-up study in Belgium from 2022 showed that the hybrid immunity group had a slower decline of antibodies than the “only vaccination” cohort [27]. In contrast to our findings, a study from 2022, conducted in Chicago, reported that antibodies against S protein did not increase significantly in a group of previously infected COVID-19 patients after the second vaccine dose (i.e., third antigen exposure) [3]. However, the antibodies were measured only 3 weeks after the first and second vaccination, which could have affected the results if the antibody responses were delayed. The second vaccine dose in previously infected COVID-19 patients may have had further immunological effects, such as broadening of the antibody response and improving the cellular immunity [3]. In the present study, non-booster-immunized PCR − participants had higher median antibodies against N protein at T2, which may indicate that they were exposed more frequently to infections than booster-immunized participants. Finally, non-vaccinated participants in our study (*n* = 14) had the lowest values of antibodies against S protein, whereas the booster-immunized (*n* = 416) had the highest values at T2. Different antibody tests, testing frequencies for SARS-CoV-2 infections, study populations (demographics and comorbidities), and vaccination coverage may explain varying results among studies [20].

The median antibody value against S protein was highest in the oldest age group (> 60 years) at T2. The older participants were more booster-vaccinated than the other age groups between T1 and T2 in our study, which might explain this finding. This observation is in line with a study from Norway among COVID-19-naïve older adults who generated good serological and cellular responses after two vaccine doses, with further improvement after three doses [28]. Interestingly, another population-based study from Norway showed that the effectiveness of booster vaccination against severe COVID-19 was lowest in the oldest age group. However, as the group was small and antibodies were not measured, a clear conclusion could not be drawn from that study [9]. Our study population had few hospitalized participants for the initial SARS-CoV-2 infection, a low comorbidity score and therefore might represent a relative healthy population with good humoral response after vaccination, also among elderly participants. We showed that when vaccination coverage increased from 0–1 to 2–4 doses, the antibody levels against S protein increased for both PCR + and PCR − participants. Hence, booster doses had an additional effect on the humoral immunity. In the present study, male participants had higher median antibodies levels against N protein at T1 and against S protein at T2. This finding should be interpreted carefully, because of the semiquantitative test for the antibodies against N protein. Both infection and vaccination induces antibodies against S protein, the possibility for detection of previous infection is therefore limited, but the use of antibodies against N protein might help to detect possible asymptomatic infections. In contrast to our study, a 2-year follow-up study from the first pandemic wave in Italy showed no significant association between sex and persistence of SARS-CoV-2 N IgG for 153 SARS-CoV-2-infected patients [18]. Although, in the multivariate analyses in our study, there was a tendency for males to be in the high spike antibody level group at T2, this finding was not statistically significant. PCR + males were older than PCR + females and older participants were more booster-immunized than younger participants, which could explain this finding. PCR + males also had higher BMI than PCR + females. Other studies have shown that older age [14, 29] and high BMI [30] are risk factors for more severe SARS-CoV-2 infection, which can result in a stronger immune response [14]. Finally, we have not found other studies which assess the association between level of education and development of antibodies after COVID-19 or vaccination. The possible explanation for the association between university degree and high antibody levels against S protein in our study could be the high level of booster vaccination among those participants, but this finding needs further assessment.

### Humoral immunity after infection, vaccination, and both

Irrespective of pre-vaccination SARS-CoV-2 infection status, the booster-immunized participants had higher antibody levels against S protein than unvaccinated and fully immunized participants. This finding is in line with a study that showed improved serological responses after booster vaccination [29]. In contrast, it has been shown that VOCs, especially the omicron variants, can reduce booster-induced immune protection by immune escape [12]. The present study’s 24-month follow-up was conducted during a time dominated by omicron variants, and booster-immunized individuals still had high levels of antibodies against S protein which might protect against infections with omicron variants. Few individuals in older age groups and the relative healthy study population is a possible explanation for high antibody levels at 24 months. Similar to our study, some studies have reported that the humoral immune response from two antigen exposures, SARS-CoV-2 infection combined with one vaccine dose or two vaccine doses among infection-naïve adults, are comparable [14, 19]. The definition we used for *fully immunized* is recommended by other authors [14, 19], but other definitions, such as receiving ≥ 3 vaccine doses, are also used [3]. Few studies consider humoral immunity after infection and vaccination-induced immunity to be equal [3]. Finally, in the present study, we had few unvaccinated participants, and the antibody persistence after infection could therefore not be assessed in this group.

### Strengths and limitations

Our study is strengthened by the relatively large, unselected sample, the prospective long-term follow-up through different VOCs, and simultaneous measurement of antibody levels against S and N protein. Another strength is the detailed and complete vaccination data provided from the national immunization registry and questionnaires. There are, however, some limitations. The questionnaire data was collected in Agder and Telemark counties in South-Eastern Norway. These results may, therefore, not be entirely representative of other areas or countries, although the region has both rural and urban areas and is considered to represent the Nordic populations well. Additionally, viral sequencing for SARS-CoV-2 was not performed; however, Norway has a national, epidemiological variant data register that identifies the dominating SARS-CoV-2 variants for the study period. Unfortunately, assessment of cell-mediated immunity or neutralizing antibodies was not feasible. Also, the antibodies against N protein were semiquantitative, which did not allow interpretation of exact antibody values. Furthermore, the present study did not assess further explanations for high versus low antibody levels at 24 months, such as host differences in genetic profiles or the type of vaccine administered. Moreover, as the study lasted up to 33 months, some participants were lost to follow-up, and recall bias may have occurred in questionnaire data. Finally, this was an observational study, with the possibility of residual confounding.

## Conclusions

Our study shows long-term benefits of vaccination for the humoral immune response in initially SARS-CoV-2-infected and SARS-CoV-2-naïve participants. Furthermore, antibody levels against spike protein among booster-immunized participants were higher than those for not booster-immunized participants, irrespective of the pre-vaccination SARS-CoV-2 infection status. This highlights the importance of booster vaccination after SARS-CoV-2 infection. Future research should assess protective humoral and cellular immunity in elderly previously infected patients, in other vulnerable patients and the timing of booster vaccines.

## Supplementary Information

 Additional file 1. Table 1 Antibodies against the SARS-CoV-2 spike (S) and nucleocapsid (N) proteins at 12 (T1) and 24 months (T2) following initial PCR testing for our fully and booster-immunized participants.

Additional file 2: Table 2 Median antibody values against spike (S) and nucleocapsid (N) protein at 12 (T1) and 24 (T2) months for the initially SARS-CoV-2 PCR + (PCR +) and initially SARS-CoV-2 PCR − (PCR −) participants stratified by sex.

Additional file 3. Fig. S1 Antibodies against the SARS-CoV-2 spike protein at 24 months for initially SARS-CoV-2-infected (PCR + , *n* = 232) and initially SARS-CoV-2-naïve (PCR − , *n* = 274) participants, stratified by vaccination status.

Additional file 4. Fig. S2 Distribution (%) of SARS-CoV-2 immunization statuses across different age groups at the 24-month follow-up.

## Data Availability

The datasets used and/or analyzed during the current study are available from the corresponding author on reasonable request.

## Abbreviations

BMI: Body mass index
CI: Confidence interval
COVID-19: Coronavirus disease
IQR: Interquartile range
mRNA: Messenger ribonucleic acid
N: Nucleocapsid
OR: Odds ratio
PCR: Polymerase chain reaction
SARS-CoV-2: Severe acute respiratory syndrome coronavirus 2
S: Spike
SYSVAK: The National Immunisation Registry (Nasjonalt vaksinasjonsregister)
VoC: Variants of concern

## References

[1] WHO. WHO coronavirus (COVID-19) dashboard World Health Organization 2025 data.who.int, WHO coronavirus (COVID-19) dashboard > cases [Dashboard]; 2025. https://data.who.int/dashboards/covid19/cases: WHO [.

[2] Fiolet T, Kherabi Y, MacDonald CJ, Ghosn J, Peiffer-Smadja N. Comparing COVID-19 vaccines for their characteristics, efficacy and effectiveness against SARS-CoV-2 and variants of concern: a narrative review. Clin Microbiol Infect. 2022;28:202–21. 34715347 10.1016/j.cmi.2021.10.005 PMC8548286

[3] Uprichard SL, O'Brien A, Evdokimova M, Rowe CL, Joyce C, Hackbart M, et al. Antibody response to SARS-CoV-2 infection and vaccination in COVID-19-naïve and experienced individuals. Viruses. 2022;14. 10.3390/v14020370 PMC8878640 35215962

[4] Altawalah H. Antibody responses to natural SARS-CoV-2 infection or after COVID-19 vaccination. Vaccines (Basel). 2021;9. 10.3390/vaccines9080910 PMC8402626 34452035

[5] Sarjomaa M, Diep LM, Zhang C, Tveten Y, Reiso H, Thilesen C, et al. SARS-CoV-2 antibody persistence after five and twelve months: a cohort study from South-Eastern Norway. PLoS ONE. 2022;17: e0264667. 35947589 10.1371/journal.pone.0264667 PMC9365168

[6] Wajnberg A, Amanat F, Firpo A, Altman DR, Bailey MJ, Mansour M, et al. Robust neutralizing antibodies to SARS-CoV-2 infection persist for months. Science. 2020;370:1227–30. 33115920 10.1126/science.abd7728 PMC7810037

[7] Dan JM, Mateus J, Kato Y, Hastie KM, Yu ED, Faliti CE, et al. Immunological memory to SARS-CoV-2 assessed for up to 8 months after infection. Science. 2021;371. 10.1126/science.abf4063 PMC7919858 33408181

[8] Bates TA, McBride SK, Leier HC, Guzman G, Lyski ZL, Schoen D, et al. Vaccination before or after SARS-CoV-2 infection leads to robust humoral response and antibodies that effectively neutralize variants. Sci Immunol. 2022;7:eabn8014. 10.1126/sciimmunol.abn8014 PMC8939472 35076258

[9] Laake I, Skodvin SN, Blix K, Caspersen IH, Gjessing HK, Juvet LK, et al. Effectiveness of mRNA booster vaccination against mild, moderate, and severe COVID-19 caused by the omicron variant in a large, population-based. Norwegian cohort J Infect Dis. 2022;226:1924–33. 36259543 10.1093/infdis/jiac419 PMC9620770

[10] Cerqueira-Silva T, de Araujo OV, Paixão ES, Júnior JB, Penna GO, Werneck GL, et al. Duration of protection of CoronaVac plus heterologous BNT162b2 booster in the omicron period in Brazil. Nat Commun. 2022;13:4154. 35851597 10.1038/s41467-022-31839-7 PMC9289933

[11] Spinardi JR, Srivastava A. Hybrid immunity to SARS-CoV-2 from infection and vaccination-evidence synthesis and implications for new COVID-19 vaccines. Biomedicines. 2023;11. 10.3390/biomedicines11020370 PMC9953148 36830907

[12] Qu P, Faraone JN, Evans JP, Zheng YM, Yu L, Ma Q, et al. Durability of booster mRNA vaccine against SARS-CoV-2 BA.2.12.1, BA.4, and BA.5 subvariants. N Engl J Med. 2022;387:1329–31. 10.1056/NEJMc2210546 PMC9511629 36069925

[13] Zheng H, Wu S, Chen W, Cai S, Zhan M, Chen C, et al. Meta-analysis of hybrid immunity to mitigate the risk of omicron variant reinfection. Front Public Health. 2024;12:1457266. 39253287 10.3389/fpubh.2024.1457266 PMC11381385

[14] Lapuente D, Winkler TH, Tenbusch M. B-cell and antibody responses to SARS-CoV-2: infection, vaccination, and hybrid immunity. Cell Mol Immunol. 2024;21:144–58. 37945737 10.1038/s41423-023-01095-w PMC10805925

[15] Van Elslande J, Oyaert M, Ailliet S, Van Ranst M, Lorent N, Vande Weygaerde Y, et al. Longitudinal follow-up of IgG anti-nucleocapsid antibodies in SARS-CoV-2 infected patients up to eight months after infection. J Clin Virol. 2021;136: 104765. 33636554 10.1016/j.jcv.2021.104765 PMC7891078

[16] L’Huillier AG, Meyer B, Andrey DO, Arm-Vernez I, Baggio S, Didierlaurent A, et al. Antibody persistence in the first 6 months following SARS-CoV-2 infection among hospital workers: a prospective longitudinal study. Clin Microbiol Infect. 2021;27:784.e1-8.e8. 33482352 10.1016/j.cmi.2021.01.005 PMC7816882

[17] Dodge MC, Ye L, Duffy ER, Cole M, Gawel SH, Werler MM, et al. Kinetics of SARS-CoV-2 serum antibodies through the alpha, delta, and omicron surges among vaccinated health care workers at a Boston hospital. Open Forum Infect Dis. 2023;10:ofad266. 10.1093/ofid/ofad266 PMC10314714 37396669

[18] Peghin M, De Martino M, Palese A, Chiappinotto S, Fonda F, Gerussi V, et al. Antibody response and risk of reinfection over 2 years among the patients with first wave of COVID-19. Clin Microbiol Infect. 2024;30:522–30. 38141821 10.1016/j.cmi.2023.12.017

[19] Krammer F, Srivastava K, Alshammary H, Amoako AA, Awawda MH, Beach KF, et al. Antibody responses in seropositive persons after a single dose of SARS-CoV-2 mRNA vaccine. N Engl J Med. 2021;384:1372–4. 33691060 10.1056/NEJMc2101667 PMC8008743

[20] Pilz S, Theiler-Schwetz V, Trummer C, Krause R, Ioannidis JPA. SARS-CoV-2 reinfections: overview of efficacy and duration of natural and hybrid immunity. Environ Res. 2022;209: 112911. 35149106 10.1016/j.envres.2022.112911 PMC8824301

[21] Sarjomaa M, Zhang C, Tveten Y, Kersten H, Reiso H, Eikeland R, et al. Risk factors for SARS-CoV-2 infection: a test-negative case–control study with additional population controls in Norway. BMJ Open. 2024;14: e073766. 38191258 10.1136/bmjopen-2023-073766 PMC10806780

[22] Tunheim G, Fossum E, Robertson AH, Rø GØI, Chopra A, Vaage JT, et al. Characterization of the SARS-CoV-2 antibody landscape in Norway in the late summer of 2022: high seroprevalence in all age groups with patterns of primary omicron infection in children and hybrid immunity in adults. BMC Infect Dis. 2024;24:841. 39164637 10.1186/s12879-024-09670-w PMC11334563

[23] Norwegian Immunisation Registry (SYSVAK). Norwegian Institute of Public Health. https://www.fhi.no/va/sysvak/.

[24] Abrahamsen R, Fell AK, Svendsen MV, Andersson E, Torén K, Henneberger PK, et al. Association of respiratory symptoms and asthma with occupational exposures: findings from a population-based cross-sectional survey in Telemark. Norway BMJ Open. 2017;7: e014018. 28336744 10.1136/bmjopen-2016-014018 PMC5372104

[25] Caspersen IH, Magnus P, Trogstad L. Excess risk and clusters of symptoms after COVID-19 in a large Norwegian cohort. Eur J Epidemiol. 2022;37:539–48. 35211871 10.1007/s10654-022-00847-8 PMC8872922

[26] Loesche M, Karlson EW, Talabi O, Zhou G, Boutin N, Atchley R, et al. Longitudinal SARS-CoV-2 nucleocapsid antibody kinetics, seroreversion, and implications for seroepidemiologic studies. Emerg Infect Dis. 2022;28(9):1859–62.26. 10.3201/eid2809.220729 PMC9423917 35868337

[27] Decru B, Van Elslande J, Steels S, Van Pottelbergh G, Godderis L, Van Holm B, et al. IgG anti-spike antibodies and surrogate neutralizing antibody levels decline faster 3 to 10 months after BNT162b2 vaccination than after SARS-CoV-2 10.3389/fimmu.2022.909910 PMC9241488 35784321

[28] Ravussin A, Robertson AH, Wolf AS, Blix K, Kjønstad IF, Solum G, et al. Determinants of humoral and cellular immune responses to three doses of mRNA SARS-CoV-2 vaccines in older adults: a longitudinal cohort study. Lancet Healthy Longev. 2023;4:e188–99. 37148891 10.1016/S2666-7568(23)00055-7 PMC10156136

[29] Wanhella KJ, Fernandez-Patron C. Biomarkers of ageing and frailty may predict COVID-19 severity. Ageing Res Rev. 2022;73: 101513. 34838734 10.1016/j.arr.2021.101513 PMC8611822

[30] Cao P, Song Y, Zhuang Z, Ran J, Xu L, Geng Y, et al. Obesity and COVID-19 in adult patients with diabetes. Diabetes. 2021;70:1061–9. 33597204 10.2337/db20-0671

