# Supplementary material for: SARS-CoV-2-specific humoral immunity in a Norwegian cohort between 2020 and 2023
Source: BMC Med. 2025 Jun 3;23:332. doi: 10.1186/s12916-025-04171-2 (PMC12135409; doi:10.1186/s12916-025-04171-2)
Supplement: Supplementary file 1 — Additional file 1. Table 1 Antibodies against the SARS-CoV-2 spike (S) and nucleocapsid (N) proteins at 12 (T1) and 24 months (T2) following initial PCR testing for our fully and booster-immunized participants. [file 12916_2025_4171_MOESM1_ESM.docx]

|  | **Vaxzevria (ASZ)** | **mRNA (Comirnaty/ Spikevax** | ***p* value^*^** |
| --- | --- | --- | --- |
| **T1** | n = 25 | n = 251 |  |
| S antibodies U/mL | 369 (80–1308) | 212 (56–1902) | 0.810 |
| N antibodies COI | 8.7 (3.4–25.7) | 12.6 (3.1–43.0) | 0.316 |
| **T2** | n = 50 | n = 415 |  |
| S antibodies U/mL | 10285 (3434–25,755) | 10,930 (5178–28,553) | 0.267 |
| N antibodies COI | 8.9 (1.6–35.1) | 13.7 (2.3–57.9) | 0.210 |

COI; cutoff index

^*^Statistical significance was defined as *p* < 0.05.

**Additional Table 1.** Antibodies against the SARS-CoV-2 spike (S) and nucleocapsid (N) proteins at 12 (T1) and 24 months (T2) following initial PCR testing for our fully- and booster-immunized participants: Vaxzevria (ASZ) AstraZeneca; ChAdOx nCoV-19; AZD1222), and Comirnaty (Pfizer/BioNTech; BNT162b2), either alone or combined with Spikevax (Moderna; mRNA-1273)
