# Supplementary material for: SARS-CoV-2-specific humoral immunity in a Norwegian cohort between 2020 and 2023
Source: BMC Med. 2025 Jun 3;23:332. doi: 10.1186/s12916-025-04171-2 (PMC12135409; doi:10.1186/s12916-025-04171-2)
Supplement: Supplementary file 2 — Additional file 2: Table 2 Median antibody values against spike (S) and nucleocapsid (N) protein at 12 (T1) and 24 (T2) months for the initially SARS-CoV-2 PCR + (PCR +) and initially SARS-CoV-2 PCR − (PCR −) participants stratified by sex. [file 12916_2025_4171_MOESM2_ESM.docx]

| **Additional Table 2** Median antibody values against spike (S) and nucleocapsid (N) protein at 12 (T1) and 24 (T2) months for the initially SARS-CoV-2 PCR+ (PCR+) and initially SARS-CoV-2 PCR- (PCR-) participants stratified by sex | | | | | | | | | | | |
| --- | --- | --- | --- | --- | --- | --- | --- | --- | --- | --- | --- |
|  | | | **PCR+** | | | | **p-value**^*^ | **PCR-** | | | **p-value^*^** |
|  |  |  | n | | Median (IQR) | |  | n | | Median (IQR) |  |
| **T1^**^**, n=287 | | | | | | | | | | | |
| S antibody  U/mL^†^ | Male | 142 | | 195 (54-996) | | 0.122 | | Na | | | |
|  | Female | 145 | | 229 (58-2921) | |  |  |  |  |  |  |
| N antibody COI^‡^ | Male | 142 | | 19.2 (4.1-50.8) | | **0.005** | |  |  |  |  |
|  | Female | 145 | | 9.4 (2.6-28.5) | |  |  |  |  |  |  |
| **T2^§^**, n=233 | | | | | | | | | | | |
| S antibody U/mL^†^ | Male | 119 | | 11178 (5658-25938) | | **0.003** | | 72 | 18381.5 (4320.5 -35131) | | 0.201 |
|  | Female | 114 | | 7562 (4200-16461) | |  |  | 175 | 10690 (3892-32032) | |  |
| N antibody  COI^‡^ | Male | 119 | | 39.5 (7.0-88.4) | | 0.541 | | 72 | 6.2 (0.9-23.3) | | 0.679 |
|  | Female | 114 | | 24.9 (4.2-102.0) | |  |  | 175 | 6.6 (0.1-22.6) | |  |

IQR: interquartile range Na: not available; U/mL: Units/milliliter; COI; cutoff index ^*^p-value <0.05; ^**^Data included all the initially SARS-CoV-2 PCR+ participants who measured antibodies at 12 months; ^†^cut off for U/mL was >0.8 U/mL; ^‡^ cut-off-index for COI was >1.0 for positive; ^§^Data included all the initially SARS-CoV-2 PCR+ participants who measured antibodies at 24-months.
