# Supplementary figures and images for: SARS-CoV-2-specific humoral immunity in a Norwegian cohort between 2020 and 2023
Source: BMC Med. 2025 Jun 3;23:332. doi: 10.1186/s12916-025-04171-2 (PMC12135409; doi:10.1186/s12916-025-04171-2)

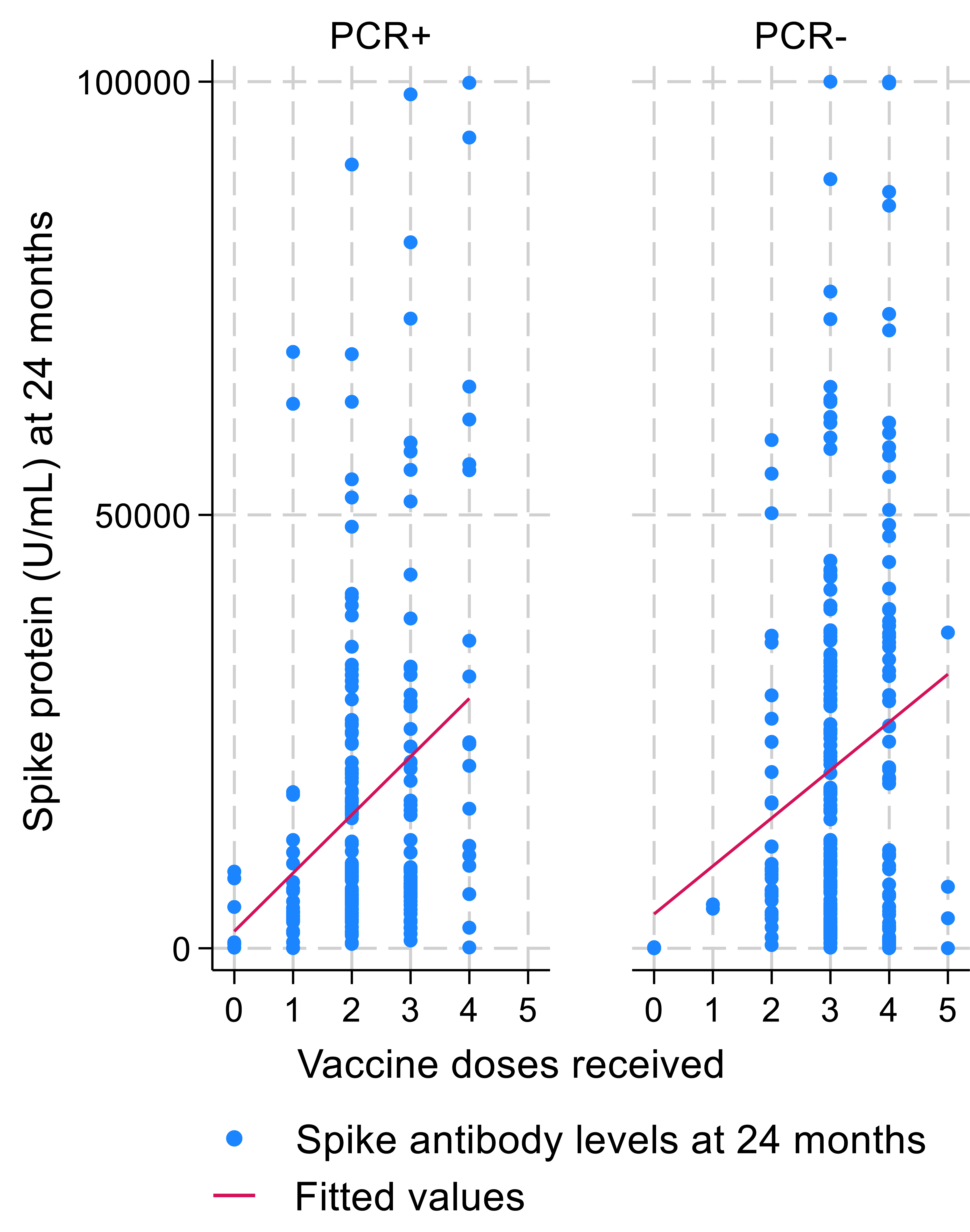

Supplement: Supplementary file 3 — Additional file 3. Fig. S1 Antibodies against the SARS-CoV-2 spike protein at 24 months for initially SARS-CoV-2-infected (PCR + , n = 232) and initially SARS-CoV-2-naïve (PCR − , n = 274) participants, stratified by vaccination status. [file 12916_2025_4171_MOESM3_ESM.tif]

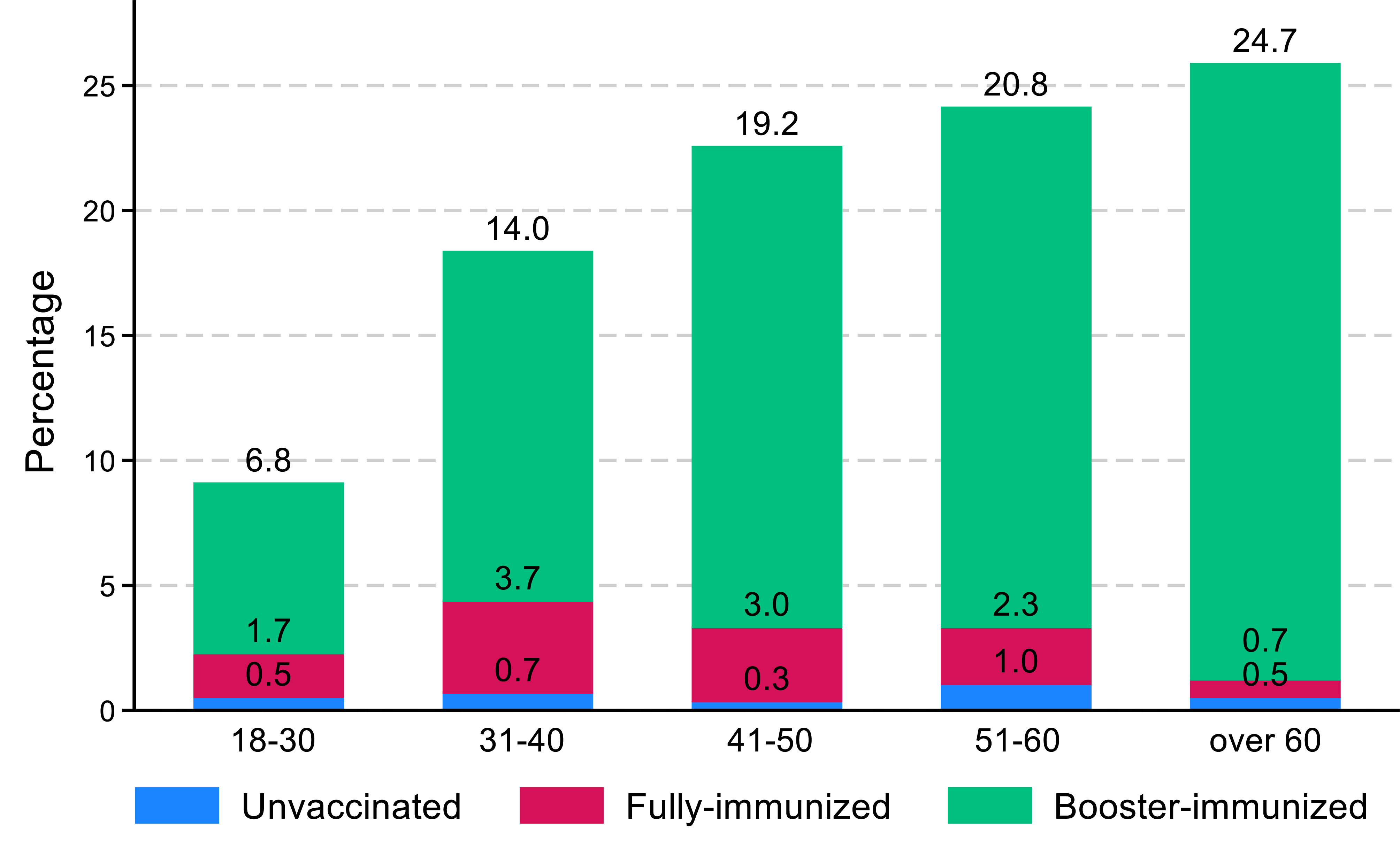

Supplement: Supplementary file 4 — Additional file 4. Fig. S2 Distribution (%) of SARS-CoV-2 immunization statuses across different age groups at the 24-month follow-up. [file 12916_2025_4171_MOESM4_ESM.tif]
